# Longer duration of initial invasive mechanical ventilation is still a crucial risk factor for moderate-to-severe bronchopulmonary dysplasia in very preterm infants: a multicentrer prospective study

**DOI:** 10.1007/s12519-022-00671-w

**Published:** 2023-01-05

**Authors:** Cong Dou, Yong-Hui Yu, Qing-Cui Zhuo, Jian-Hong Qi, Lei Huang, Yan-Jie Ding, De-Juan Yang, Li Li, Dan Li, Xiao-Kang Wang, Yan Wang, Xin Qiao, Xiang Zhang, Bing-Jin Zhang, Hai-Yan Jiang, Zhong-Liang Li, Simmy Reddy

**Affiliations:** 1 Department of Neonatology, Shandong Provincial Hospital, Shandong University, Jinan, 250021 China; 2 Department of Neonatology, Shandong Provincial Maternal and Child Health Care Hospital, Jinan, 250014 China; 3 Department of Neonatology, Shandong Provincial Hospital affiliated to Shandong First Medical University, Jinan, 250021 China; 4 Department of Neonatology, Qilu Hospital of Shandong University, Jinan, 250012 China; 5 Department of Neonatology, Yantai Yuhuangding Hospital, Yantai, 264000 China; 6 Department of Neonatology, The First Affiliated Hospital of Shandong First Medical University, Jinan, 250014 China; 7 Department of Neonatology, Linyi People’s Hospital, Linyi, 276000 China; 8 Department of Neonatology, Liaocheng People’s Hospital, Liaocheng, 252000 China; 9 Department of Neonatology, The Affiliated Taian City Central Hospital of Qingdao University, Taian, 271000 China; 10 Department of Neonatology, Jinan Maternity and Child Healthcare Hospital, Jinan, 250001 China; 11 Department of Neonatology, Hebei Petro China Central Hospital, Langfang, 065000 China; 12 Department of Neonatology, Shengli Olifield Central Hospital, Dongying, 257000 China; 13 Department of Neonatology, The Third Hospital of Baogang Group, Baotou, 014000 China; 14 Department of Neonatology, W.F. Maternal and Child Health Hospital, Weifang, 261011 China; 15 Cheeloo College of Medicine, Shandong University, Jinan, 250000 China

**Keywords:** Bronchopulmonary dysplasia, Invasive mechanical ventilation, Multicenter cohort, Preterm infants, Prospective

## Abstract

**Objectives:**

We aimed to evaluate the risk factors for moderate-to-severe bronchopulmonary dysplasia (BPD) and focus on discussing its relationship with the duration of initial invasive mechanical ventilation (IMV) in very preterm neonates less than 32 weeks of gestational age (GA).

**Methods:**

We performed a prospective cohort study involving infants born at 23–31 weeks of GA who were admitted to 47 different neonatal intensive care unit (NICU) hospitals in China from January 2018 to December 2021. Patient data were obtained from the Sina-northern Neonatal Network (SNN) Database.

**Results:**

We identified 6538 very preterm infants, of whom 49.5% (3236/6538) received initial IMV support, and 12.6% (823/6538) were diagnosed with moderate-to-severe BPD symptoms. The median duration of initial IMV in the moderate-to-severe BPD group was 26 (17–41) days, while in the no or mild BPD group, it was 6 (3–10) days. The incidence rate of moderate-to-severe BPD and the median duration of initial IMV were quite different across different GAs. Multivariable logistic regression analysis showed that the onset of moderate-to-severe BPD was significantly associated with the duration of initial IMV [adjusted odds ratio (AOR): 1.97; 95% confidence interval (CI): 1.10–2.67], late-onset neonatal sepsis (LONS), and patent ductus arteriosus (PDA).

**Conclusion:**

In this multicenter cohort study, the duration of initial IMV was still relatively long in very premature infants, and the longer duration of initial IMV accounts for the increased risk of moderate-to-severe BPD.

## Background

With the increasing survival rates of preterm infants, the incidence of bronchopulmonary dysplasia (BPD) has remained steady or increasing [1, 2]. Moderate-to-severe BPD brings about serious respiratory morbidity and neurosensory impairment, thus impacting the quality of life in affected pediatric and even adult patients [3, 4]. BPD was first described in 1967 and is considered the result of over-aggressive invasive mechanical ventilation (IMV) of relatively mature lungs (≥ 32 weeks of gestation) with an excessively high peak pressure of the supplemental gas mixture containing a higher proportion of oxygen and lacking surfactant [5]. However, the histology and pathogenesis of BPD have changed with a rise in the survival rates of premature infants at smaller gestational ages and weight. In 2001, the National Institute of Child Health and Human Development (NICHD) proposed the “New BPD” based on the severity and postmenstrual age (PMA), which is used most widely now [6]. Moreover, the NICHD Neonatal Research Network proposed a new evidence-based and severity-based definition of BPD in 2018 [7, 8]. The new definition of BPD focuses more on alveolar developmental arrest; nevertheless, whether the application status of initial invasive mechanical ventilation (IMV) still plays an important role in the occurrence and development of BPD still deserves our attention.

Series of guidelines such as the 2014 American Academy of Pediatrics guidelines [9] and 2019 European consensus guidelines [10] on the management of respiratory distress syndrome highly recommend the early use of pulmonary surfactant and the avoidance and minimization of IMV. We established the Sina-northern Neonatal Network (SNN) database and its online website (www.snn-med.com), and our previous study showed that about 41.9% of preterm infants [< 32 weeks of gestational age (GA)] received initial IMV. The incidence of BPD in the noninvasive ventilation failure group of very preterm infants can reach up to 37.6% [11]. The difference is statistically significant compared with that of the successful noninvasive ventilation receiving subjects, suggesting that the respiratory management of preterm infants may be related to the occurrence of BPD. In recent years, with the improvement of the treatment ability of very preterm infants, an increasing number of infants with smaller GAs and birth weight survive, and we urgently need multicenter large-scale research to reassess the effect of iatrogenic factors, especially the duration of initial IMV, in addition to alveolar arrest, on the development of moderate-to-severe BPD.

The main objectives of this study were to (1) investigate the incidence of moderate-to-severe BPD and the duration of initial IMV for different GAs in very preterm infants and (2) analyze the prenatal and in-hospital characteristics and identify the risk factors of moderate-to-severe BPD in very preterm neonates, especially its relationship with the duration of initial IMV.

## Methods

### Data source and study population

We performed a multicenter prospective cohort study of very preterm infants (GA: 23–31 weeks) in 47 neonatal intensive care units (NICUs) of level II or level III in China from January 1, 2018, through December 31, 2021. Patient records were obtained from the SNN database. SNN is a clinical research database in China and has strict data entry and quality-control standards. Trained data abstractors prospectively collected infant information at each NICU and electronically shared it each time among all provincial NICUs. All 47 hospitals collected data on very preterm infants using the SNN database with standardized definitions during the three-year study period. Written informed consents were obtained from all parents of the infants at each participating institution. Data on live-born infants were collected prospectively until discharge or death. Infants whose data were missing, with severe congenital heart and lung malformations, who transferred out, and those who died before 36 weeks of PMA were excluded from this study. The patterns of the initial IMV included both pressure support ventilation and volume control mode.

### Study definitions

BPD was defined as the use of supplemental oxygen for more than 28 days of life and further stratified as mild, moderate, or severe according to the NICHD consensus-2001 [6]. Moderate-to-severe BPD was defined as any infant requiring continuous supplemental oxygen at 36 weeks of PMA or at hospital discharge, whichever occurred earlier. Small for gestational age (SGA) was defined as birth weight less than the 10th percentile for GA based on the Fenton growth curve [12]. Neonatal respiratory distress syndrome (NRDS) was diagnosed based on the presence of signs of respiratory distress in a typical chest X-ray and/or the need for surfactant. The patent ductus arteriosus (PDA) was diagnosed depending on signs of a murmur, bounding pulses, active precordium, and echocardiography. The presence of intraventricular hemorrhage (IVH) symptoms was diagnosed by evaluating a head ultrasound performed before 14 days of life and graded according to the Papile classification [13]. The diagnosis of necrotizing enterocolitis (NEC) was performed based on Bell’s modified staging criteria. Early-onset neonatal sepsis (EONS) was defined as premature rupture of the placental membrane more than 18 h before delivery, infectious disease development in neonates within 72 h of live birth, and abnormal values for two or more nonspecific infection indicators. If blood or cerebrospinal fluid (CSF) culture was positive for microbial infections, culture-positive septicaemia was diagnosed [14, 15]. Late-onset neonatal sepsis (LONS) was diagnosed by the clinical manifestations of systemic infection after three days of birth as well as abnormal values for two or more of the following nonspecific infection indicators: white blood cell (WBC) count < 5 × 10^9^/L or > 20 × 10^9^/L, C-reactive protein (CRP) ≥ 10 mg/L, platelets (PLTs) ≤ 100 × 10^9^/L, and procalcitonin (PCT) > 2 ng/mL. Likewise, if the blood or CSF culture was positive, culture-positive septicaemia was diagnosed [16].

### Statistical analysis

SPSS software version 25 (IBM Corp., NY, USA) and SAS version 9.3 software (SAS Institute, Inc., Cary, NC) were used for data analysis. Categorical variables are presented as percentages, and numerical data are presented as medians with 25th and 75th percentiles (interquartile range, IQR) and were compared by rank-sum tests. Standard descriptive analyses of the demographic and clinical data were performed by using Student’s *t* test, Mann–Whitney *U* test, or chi-squared (*χ*2) test, as appropriate. A *P* value of less than 0.05 was considered statistically significant. The odds ratio (OR) was calculated using multivariable logistic regression (MLR), with adjustment for factors that had a *P* value of < 0.05 in the univariable analyses. The MLR model, including all covariates, allowed us to assess the independent risk for the primary outcome of the individual patient. Receiver operating characteristic (ROC) curve analysis was performed to determine the cut-off value for continuous variables. The area under the ROC curve (AUC) was considered a measure of cut-off point accuracy. AUC values ≥ 0.90, 0.80–0.89, 0.70–0.79, and < 0.70 were considered excellent, good, reasonable, and poor, respectively.

## Results

**Fig. 1:**
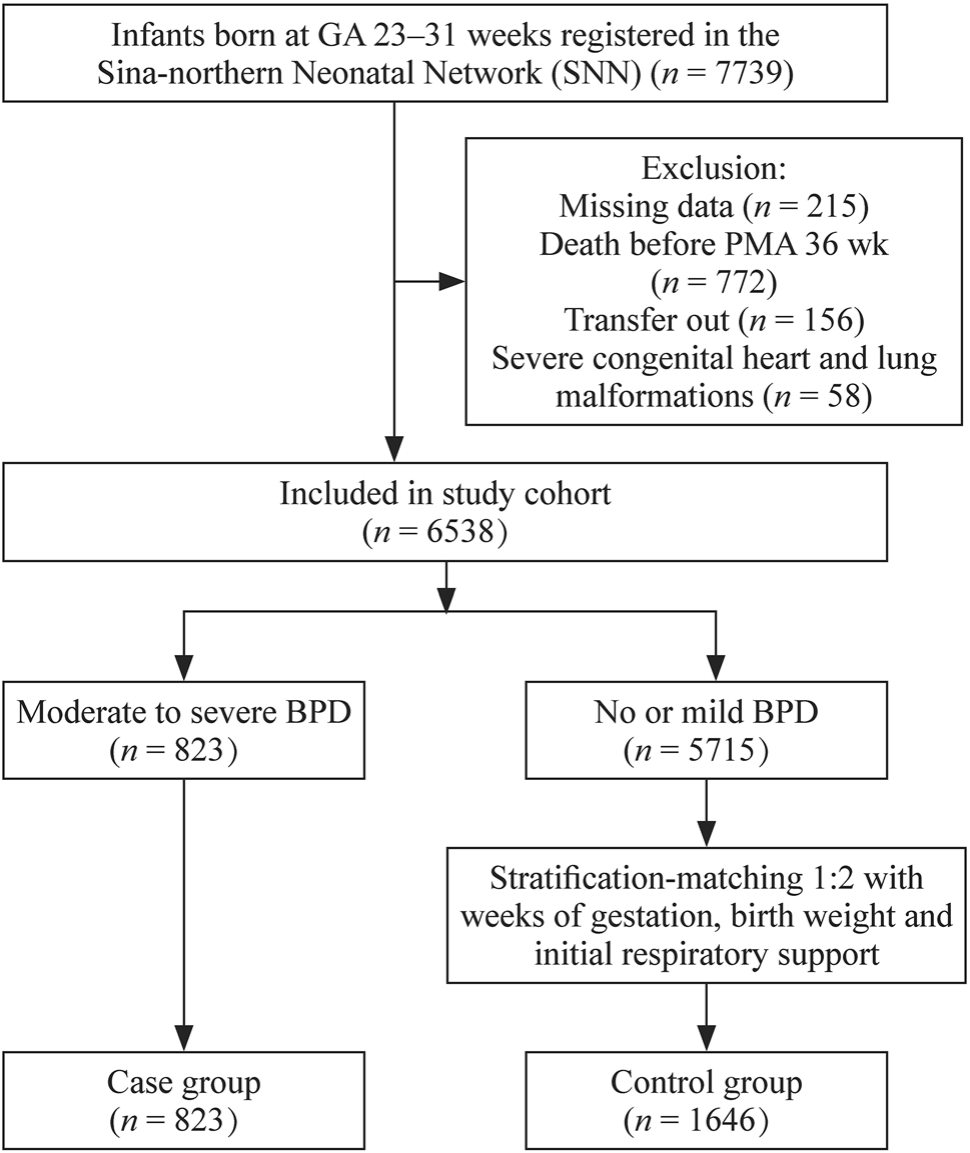
Study participants

**Fig. 2:**
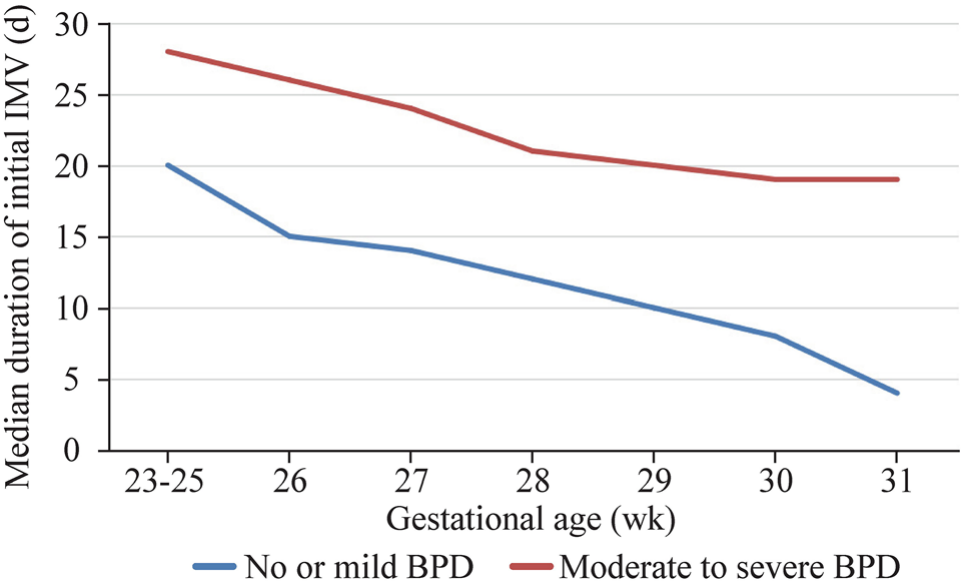
Median duration of initial IMV (days) in different gestational age

After excluding ineligible subjects, the study cohort finally included 6538 preterm infants with GA ranging from 23 to 31 weeks who were born at 47 different Chinese hospitals. Of these infants, 823 (12.6%) developed moderate-to-severe BPD, while 5715 (87.4%) were classified as having no or mild BPD (Fig. 1). The incidences of moderate-to-severe BPD in very preterm infants showed a decreasing trend with increasing GA, from 68.4% (13/19) at 23–24 weeks to 3.9% (74/1875) at 31 weeks of GA (Table 1).About 49.5% (3236/6538) of patients required initial IMV support, while that in moderate-to-severe BPD cases was 89.1% (733/823). The median duration of initial IMV in the moderate-to-severe BPD group was 26 (17–41) days and that in the no or mild BPD group was 6 (3–10) days. There were significant differences between the median duration of initial IMV support between moderate-to-severe and no or mild BPD groups as well as among different GAs. The median duration of initial IMV of GA at 23–31 weeks ranged from 19 to 28 days in the moderate-to-severe BPD group (Fig. 2).

**Table 1:** Incidence of moderate-to-severe BPD among infants born at 23–31-week gestation

Gestational age (wk)	Total cases	No or mild BPD (%)	Moderate-to-severe BPD (%)
23–24	19	6 (36.8)	13 (68.4)
25	87	42 (66.5)	45 (51.7)
26	430	273 (63.5)	157 (36.5)
23–26	536	321 (59.9)	215 (40.1)
27	539	364 (67.5)	175 (32.5)
28	675	553 (81.9)	122 (18.1)
29	1306	1187 (90.9)	119 (9.1)
30	1607	1489 (92.7)	118 (7.3)
31	1875	1801 (96.1)	74 (3.9)
Total	6538	5715 (87.4)	823 (12.6)

*BPD* bronchopulmonary dysplasia

All the included infants were allocated to the moderate-to-severe and no or mild BPD groups. The baseline characteristics of these subjects are described in Table 2. Among the infants with moderate-to-severe BPD, the median GA was 27.3 (26.1–28.6) weeks, and birth weight (BW) was 862 (780–940) g. Among them, 733 (89.1%) infants received initial support of IMV, and 744 (90.4%) had an initial fraction of inspired oxygen (FiO2) ≥ 30%. Compared with the no or mild BPD group, infants in the moderate-to-severe BPD group had lower GA and BW. There were statistically significant differences between these two groups in cesarean section, chorioamnionitis, male sex, SGA, APGAR scores for one and five minutes, surfactant treatment, initial FiO2 ≥ 30%, the median duration of initial IMV (days), the median duration of initial noninvasive ventilation, and postnatal dexamethasone treatment (Table 2).

**Table 2:** Characteristics of infants before and after 1:2 match with GA, BW and initial respiratory support

Variables	Full cohort			Matched cohort		
	Moderate-to-severe BPD group (*n* = 823)	No or mild BPD group (*n* = 5715)	*P* value	Case group (*n* = 823)	Control group (*n* = 1646)	*P* value
Maternal characteristics						
Maternal age, (IQR), y	32 (29, 36)	31 (28, 35)	0.101	32 (29, 36)	31 (29, 35)	0.649
Spontaneous pregnancy, *n* (%)	577 (70.1)	4172 (73)	0.142	577 (70.1)	1213 (73.7)	0.347
Cesarean section, *n* (%)	500 (60.8)	3886 (68.0)	0.043	500 (60.8)	1045 (63.5)	0.214
DM or GDM, *n* (%)	116 (14.1)	732 (12.8)	0.150	116 (14.1)	202 (12.3)	0.282
Chorioamnionitis, *n* (%)	48 (5.8)	183 (3.2)	0.022	48 (5.8)	77 (4.7)	0.421
Antenatal corticosteroid, *n* (%)	751 (91.2)	5258 (92.0)	0.536	751 (91.2)	1519 (92.3)	0.460
PROM ≥ 24 h, *n* (%)	145 (17.6)	909 (15.9)	0.327	145 (17.6)	253 (15.4)	0.244
Gestational hypertension, *n* (%)	156 (18.9)	886 (15.5)	0.241	156 (18.9)	277 (16.8)	0.193
Neonatal characteristics						
GA, median (IQR), wk	27.3 (26.1–28.6)	29.2 (28.1–30.2)	< 0.001	27.3 (26.1–28.6)	27.3 (26.0–28.6)	1.000
Birth weight, median (IQR), g	862 (780–940)	1214 (1130–1350)	< 0.001	862 (780–940)	862 (782–950)	1.000
Male, *n* (%)	478 (58.1)	2875 (50.3)	< 0.001	478 (58.1)	858 (52.1)	0.042
SGA, *n* (%)	77 (9.3)	394 (6.9)	< 0.01	77 (9.3)	133 (8.1)	0.354
Multiple births, *n* (%)	170 (20.7)	1120 (19.6)	0.552	170 (20.7)	314 (19.1)	0.786
Apgar 1 min score ≤ 7, *n* (%)	661 (80.3)	2223 (38.9)	< 0.001	661 (80.3)	1253 (76.1)	0.562
Apgar 5 min score ≤ 7, *n* (%)	443 (53.8)	1103 (19.3)	< 0.001	443 (53.8)	835 (50.7)	0.343
Treatment and complications during hospitalization						
Surfactant treatment, *n* (%)	741 (90.0)	4652 (81.4)	0.034	741 (90.0)	1455 (88.4)	0.454
NRDS, *n* (%)	789 (95.9)	5201 (91.0)	0.172	789 (95.9)	1542 (93.7)	0.614
Caffeine, *n* (%)	787 (95.6)	5109 (89.4)	0.096	787 (95.6)	1532 (93.1)	0.682
Postnatal dexamethasone treatment, *n* (%)	451 (54.8)	720 (12.6)	< 0.001	451 (54.8)	500 (30.4)	< 0.001
Initial invasive respiratory support, *n* (%)	733 (89.1)	2650 (46.4)	< 0.001	733 (89.1)	1465 (89.0)	1.000
Initial FiO_2_ ≥ 30%, *n* (%)	744 (90.4)	3006 (52.6)	< 0.001	744 (90.4)	1460 (88.7)	0.653
Initial IMV duration, median (IQR), d	26 (17–41)	6 (3–10)	< 0.001	26 (17–41)	14 (9–17)	< 0.001
Initial noninvasive ventilation, median (IQR), d	31 (24–44)	14 (10–20)	< 0.001	31 (24–44)	22 (17–26)	< 0.001
Early-onset neonatal sepsis, *n* (%)	165 (20.0)	680 (11.9)	< 0.001	165 (20.0)	295 (17.9)	0.231
Late-onset neonatal sepsis, *n* (%)	370 (44.9)	1434 (25.1)	< 0.001	370 (44.9)	448 (27.2)	< 0.001
PDA, *n* (%)	560 (68.0)	2337 (40.9)	< 0.001	560 (68.0)	744 (45.2)	< 0.001
IVH grade 3 or 4, *n* (%)	126 (15.3)	377 (6.6)	< 0.001	126 (15.3)	146 (8.9)	0.042
NEC stage 2 or 3, *n* (%)	34 (4.1)	206 (3.6)	0.680	34 (4.1)	63 (3.8)	0.910
Pneumothorax, *n* (%)	20 (2.4)	114 (2.0)	0.741	20 (2.4)	35 (2.1)	0.865
Pulmonary hemorrhage, *n* (%)	86 (10.4)	297 (5.2)	0.023	86 (10.4)	142 (8.6)	0.091
ROP ≥ grade 3, *n* (%)	114 (13.9)	257 (4.5)	< 0.001	114 (13.9)	76 (4.6)	< 0.001
Length of stay, *n* (%)	95 ± 8.2	36 ± 7.4	< 0.001	95 ± 8.2	76 ± 7.6	< 0.001
GA at discharge median (IQR), wk	41.8 (39.6–42.7)	36.2 (35.1–37.8)	< 0.001	41.8 (39.6–42.7)	38.5 (37.2–39.6)	< 0.001
Weight at discharge median (IQR), g	2350 (2245–2460)	2095 (2020–2180)	< 0.001	2350 (2245–2460)	2145 (2045–2260)	0.020

*BPD* bronchopulmonary dysplasia, *GA* gestational age, *BW* birth weight, *IQR* interquartile range, *SGA* small for gestational age, *PROM* premature rupture of membranes, *GDM* gestational diabetes mellitus, *DM* diabetes mellitus, *NRDS* neonatal respiratory distress syndrome, *FiO*_*2*_ fraction of inspired oxygen, *IMV* invasive mechanical ventilation, *PDA* patent ductus arteriosus, *IVH* intraventricular hemorrhage, *NEC* necrotizing enterocolitis, *ROP* retinopathy of prematurity

The 6538 subjects were divided into two groups, namely, the case group and the control group, at 1:2 stratification (case group *n* = 823, control group *n* = 1646; Fig. 1) matched with GA (± 1 week), BW (± 100 g), and mode of initial respiratory support (initial IMV or initial noninvasive ventilation). Characteristics of GA, BW, and initial respiratory support were completely matched between the two groups. At the same time, there were no differences in cesarean section, chorioamnionitis, SGA, or APGAR scores (all *P* > 0.05). Notably, there was a significant difference in gender between the two groups (*P* < 0.05). Following stratification matching, neonates in the case group had significantly higher incidence rates of LONS, IVH grade 3–4, PDA, and retinopathy of prematurity (ROP) ≥ grade 3. They were more likely to receive postnatal dexamethasone treatment, as well as a relatively longer duration of initial IMV and noninvasive ventilation. At discharge, individuals in the case group had been hospitalized longer, had a higher body weight and were older than those in the control group (Table 2).

To evaluate the effect of these risk factors on the occurrence of moderate-to-severe BPD, we included gender, duration of initial IMV (days), duration of initial noninvasive ventilation (days), LONS, PDA, and IVH grade 3–4 for the MLR analysis (Table 3), and we adjusted the treatment of postnatal dexamethasone. Time of noninvasive ventilation, gender, and IVH grade 3–4 were found to have no correlation with the occurrence of moderate-to-severe BPD (all *P* > 0.05). The duration of initial IMV [adjusted odds ratio (AOR) = 1.97, 95% confidence interval (CI) = 1.10–2.67], LONS (AOR = 2.07, 95% CI = 1.54–3.26), and PDA (AOR = 1.45, 95% CI = 1.07–2.11) might have increased the risk of moderate-to-severe BPD onset (Table 3).

**Table 3:** Logistic regression analyses of risk factors for moderate-to-severe BPD

Risk	*P* value	Crude OR (95% CI)	Adjusted OR^a^ (95% CI)
Male	0.173	1.22 (0.87–1.58)	1.05 (0.77–1.53)
Duration of initial IMV (d)	< 0.001	1.74 (1.12–2.36)	1.97 (1.10–2.67)
Duration of initial noninvasive ventilation (d)	0.782	1.09 (0.91–1.25)	1.02 (0.82–1.55)
Late-onset neonatal sepsis	< 0.001	2.43 (2.16–4.17)	2.07 (1.54–3.26)
PDA	< 0.001	1.58 (1.13–2.41)	1.45 (1.07–2.11)
IVH grade 3 or 4	0.455	1.18 (0.95–1.65)	1.16 (0.83–1.47)

*BPD* bronchopulmonary dysplasia, *OR* odds ratio, *CI* confidence interval, *IMV* invasive mechanical ventilation, *PDA* patent ductus arteriosus, *IVH* intraventricular hemorrhage. ^a^ Multilevel mixed-effects logistic regression models were used accounting for the intracluster correlation among the infants within hospitals

**Fig. 3:**
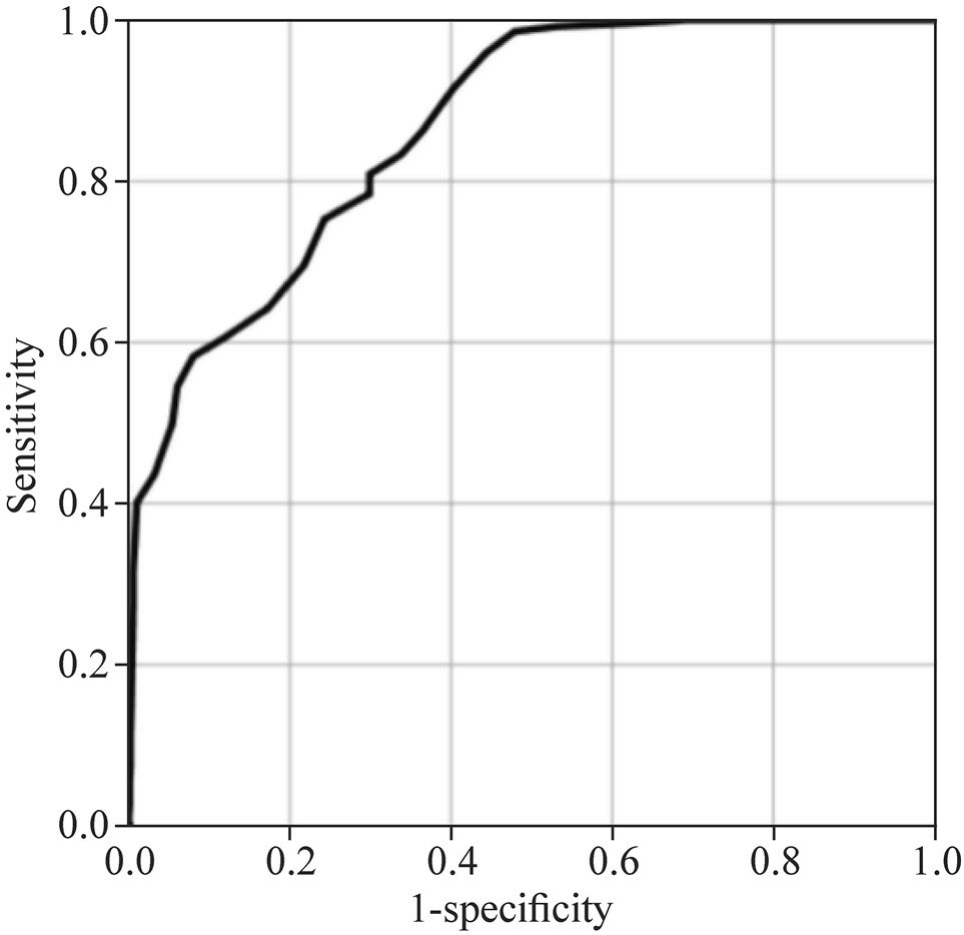
ROC curve of initial IMV time (d)

The cut-off value of the initial IMV time was set to 22 days through the ROC curve (sensitivity: 81.0%; specificity: 70.1%; area = 0.87; 95% CI: 0.74 to 0.92; *P* = 0.000) (Fig. 3). After 22 days of IMV, the risk of moderate-to-severe BPD was found to increase 1.97 times for each additional day of IMV (Table 3).

## Discussion

This cohort study included 6538 very preterm infants with GA of 23–31 weeks. Among them, 8.2% of infants had a GA of ≤ 26 weeks. We identified that 49.5% of enrolled infants were prescribed initial IMV support, and 12.6% developed moderate-to-severe BPD. The median duration of initial IMV in the moderate-to-severe BPD group was 26 (17–41) days, while in no or mild BPD, it was 6 (3–10) days. This study suggests the current status of initial IMV support and exhaustively describes the relationship between prolonged duration of initial IMV and occurrence of moderate-to-severe BPD in a large-sample prospective cohort. The findings of this investigation emphasize the severity of a longer duration of initial IMV and the urgent need for quality improvement of respiratory management in neonates, which are crucial for guiding clinical practice on the prevention of moderate-to-severe BPD in Chinese NICUs.

In the present study, the incidence rate of moderate-to-severe BPD was 12.6% in neonates born at 23–31 weeks of gestation. According to the Vermont Oxford Network (VON), about 10% of infants born with GA between 22 and 29 weeks died before 36 weeks of PMA; 36.9% developed grade 1 or 2 BPD, and 3.7% presented grade 3 BPD in the United States [3]. In total, 59% of infants were born at ≤ 26 weeks of GA, compared with 8.2% of infants in the present cohort. Restricting our data set to infants who were born at ≤ 26 weeks of GA and survived to 36 weeks of PMA, we found higher BPD rates in these two studies. Furthermore, Torchin et al. reported in the EPIPAGE-2 cohort study in France [17] that the incidence rate of moderate-to-severe BPD in preterm infants with GA of 23–31 weeks was 11.1% and that at ≤ 26 weeks of GA was 38.5%. In this cohort study, the incidence rate of moderate-to-severe BPD at ≤ 26 weeks of GA was 40.1%, indicating a relatively higher occurrence rate in our case, which deserves further attention from clinicians.

In this study, preterm infants with moderate-to-severe BPD were associated with more complicated adverse outcomes, including a longer length of hospital stay, an older GA, and greater body weight at discharge. According to the published literature, middle- and long-term follow-up of moderate-to-severe BPD showed poor health and neurodevelopmental outcomes compared with preterm peers without or with mild BPD. Schmidt et al. found that preterm infants with BPD have more than doubled odds of late death or neurodevelopmental disability when compared with preterm infants without BPD [18–20]. Moreover, Jensen et al. [3] identified that greater BPD severity could be associated with more frequent development of major neonatal morbidities, in-hospital mortality, and the use of supplemental respiratory support at discharge. Therefore, early prevention and detection of moderate-to-severe BPD are extremely important.

The American Academy of Pediatrics Guidelines-2014 for respiratory support at birth and the European Guidelines-2016 for the prevention and treatment of NRDS have strongly recommended the implementation of preventive continuous positive airway pressure (CPAP) immediately after birth with subsequent selective surfactant administration in very preterm infants; if a ventilator is needed, rapid extubation is preferred to prolonged ventilation. The proportion of very preterm infants requiring initial IMV support was 49.5% in the whole cohort and 89.1% in moderate-to-severe BPD subjects. A retrospective cohort study from Spain between 2015 and 2019 reported that the proportion of preterm infants with GA of < 32 weeks requiring initial IMV support was approximately 31.8% [21]. In this study, the median duration of initial IMV in the moderate-to-severe BPD group was 26 (17–41) days and that in the no or mild BPD group was 6 (3–10) days. Moreover, Dassios et al. reported in a whole-population study with infants at < 28 weeks of gestation who were admitted to a neonatal unit in England between 2014 and 2018 that the duration of ventilation in the BPD group was 18 (7–35) days and that in the no BPD group was 4 (2–10) days [22]. Although the median GA of our cohort was relatively larger, the initial IMV duration was longer, suggesting that prolonged IMV duration could initiate secondary health complications. Therefore, a higher proportion of initial IMV and prolonged IMV duration pinpoints that there are gaps in the implementation of international guidelines in our region, which means further quality improvement of respiratory management of premature infants is urgently needed.

The comparison of baseline data between our study and previous studies confirmed that GA, BW, and effective initial resuscitation were significant risk factors for developing moderate-to-severe BPD [23]. After matching by GA, BW, and initial respiratory support, we found that the duration of initial IMV was consistently an important disease-modulatory factor related to the onset of moderate-to-severe BPD. Studies have shown that among infants with extremely low birth weight (ELBW), a longer cumulative duration of IMV largely accounts for the increased risk of chronic respiratory diseases associated with re-initiation of IMV [24]. Despite the life-saving benefits of IMV, Choi et al. reported the downside of mechanical ventilation in inducing moderate-to-severe BPD in very preterm neonates, especially when applied for a longer duration [25]. Several studies have reported similar consequences and suggested seriously considering the actual necessity of prolonged IMV support in preterm neonates [26–28]. Likewise, our study results also suggest that clinicians should consider an optimized mechanical ventilation strategy to minimize the incidence of moderate-to-severe BPD in very preterm neonates. An efficient strategy should include weaning from the ventilator as soon as clinically possible, following early extubation, and noninvasive ventilator support whenever possible.

Furthermore, we found that LONS and PDA increased the risk of moderate-to-severe BPD, which was consistent with previous reports. In a retrospective, population-based cohort study, Lapcharoensap et al. detected a reduction in rates of nosocomial infections associated with declining rates of BPD [29]. Many studies have indicated that there could be a pathophysiological relationship between sepsis and BPD. It is particularly important to control the occurrence of LONS to reduce the rate of BPD [30, 31]. PDA has been historically implicated in the development of BPD, which may be medically treated or surgically closed [32]. Recently, several studies have confirmed that prophylactic surgical ligation of PDA could not improve the incidence of BPD onset [33]. Additional studies are warranted to determine the weightage of benefits versus risks of non-intervention for hemodynamically significant PDA in premature infants. It is suggested that clinicians first manage PDA to strengthen the prevention and control of nosocomial infections, resulting in a reduced rate of occurrence of moderate-to-severe BPD.

The major strengths of our study are as follows. This was a large prospective cohort involving very preterm infants with a GA of 23–31 weeks. This study showed the incidence of moderate-to-severe BPD and the multicenter application status of initial IMV, demonstrating the causal relationship between the incidence of moderate-to-severe BPD and the duration of initial IMV at different GAs. Furthermore, the results of this study suggest that in addition to alveolar developmental arrest of premature infants leading to “new BPD”, iatrogenic factors still affect the occurrence and development of BPD, which should not be ignored. The occurrence of moderate-to-severe BPD is still related to NICU management, especially respiratory management, implying that clinicians still need to pay attention to and optimize respiratory management in very preterm infants and shorten the duration of initial IMV as much as possible. In addition, our study was based on a clinical research database that collected data according to internationally standardized definitions. Hence, our data source was reliable and strictly quality controlled, ensuring the accuracy of the variables in this study.

However, our study also suffers from several limitations. First, the NICHD consensus in 2001 was followed to define different stages of BPD severity and to exclude those infants who died before 36 weeks of PMA, which might have included some infants with severe BPD, thereby introducing selection bias to this study. Second, we did not analyze variations in IMV applications among different NICUs. We are planning to implement a survey on initial respiratory management strategies in multicenter NICUs to identify possible reasons for the application of prolonged IMV while exploring optimized respiratory management to prevent the occurrence of moderate-to-severe BPD. In addition, the specific modes and parameters of invasive mechanical ventilation were not analyzed in this study. We plan to conduct in-depth analysis and discussion on different ventilator modes and parameters in the following study.

In conclusion, the duration of initial IMV was relatively longer in very preterm infants in this regional multicenter study. A longer duration of initial IMV was associated with significantly higher incidences of moderate-to-severe BPD. Optimization of early respiratory management in very premature infants may play an important role in reducing the occurrence of moderate-to-severe BPD, which deserves further study. Next, we will explore the possible reasons for the longer duration of initial IMV, analyze the potential influencing factors of self-exposure and NICU management, and strive to carry out continuous quality improvement of NICU management factors.

## Data Availability

The datasets generated during and/or analyzed during the current study are available from the corresponding author on reasonable request.
